# Red-green disease: overreliance on color-coded OCT RNFL analysis in glaucoma diagnosis

**DOI:** 10.1038/s41433-026-04600-3

**Published:** 2026-06-10

**Authors:** Venkata S. Jonnakuti, Misha F. Syed, Praveena Gupta

**Affiliations:** Department of Ophthalmology and Visual Sciences, University of Texas Medical Branch, Galveston, TX USA

**Keywords:** Diagnosis, Medical imaging

Optical coherence tomography (OCT) has become indispensable in glaucoma and neuro-ophthalmic care. Among its outputs, color-coded neuroretinal rim and peripapillary retinal nerve fibre layer (RNFL) maps have particular intuitive appeal: green for within normal limits, yellow for borderline and red for outside normal limits. These summaries are ubiquitous in clinical practice and often shape diagnostic confidence alongside optic disc examination, intraocular pressure and visual field testing. Yet few clinicians consider how these thresholds are derived, or whether the normative data behind them represent the diversity of patients seen in everyday practice.

Each manufacturer defines ‘normal’ RNFL thickness based on proprietary reference datasets. For the widely used Cirrus HD-OCT (Carl Zeiss Meditec), the normative database was constructed from 284 eyes across seven sites, representing 122 White, 63 Chinese, 51 African-descent and 35 Hispanic individuals [1, 2]. Participants were mostly middle-aged, with normal axial lengths and good visual acuity.

While these data were a significant achievement for their time, they represent a relatively narrow demographic and biometric range. Comparable datasets from other manufacturers, including Spectralis, Avanti and Triton, contain similarly modest sample sizes, typically a few hundred healthy eyes, often drawn from a single region or ancestry group [3–5]. These small normative cohorts form the statistical backbone of RNFL colour coding. Each measured sector is compared with an age-matched distribution from the reference sample and percentile cut-offs determine the colour displayed on screen.

This process assumes that the reference population accurately reflects biological diversity across ancestry, ocular biometry and age. Yet the simplicity of the colour map conceals this assumption. The risk arises not from the imaging technology itself but from the limited generalisability of the data on which its interpretive cues depend.

A growing body of literature demonstrates that mean RNFL thickness and optic disc morphology vary with ancestry, axial length and disc size [6–10]. Individuals of African or Asian descent may, on average, have thinner temporal RNFLs or larger discs than those of European ancestry and high myopia is associated with diffuse thinning across multiple sectors. When such physiologic differences are under-represented in a normative dataset, the resulting percentile boundaries can misclassify healthy eyes as abnormal or mask early damage in others.

Consider a highly myopic patient or someone with an unusually large optic disc: the RNFL map may show red or yellow sectors, not because of true pathology but because their baseline anatomy lies outside the narrow range of the original reference population. Conversely, an eye with small discs and naturally thicker nerve fibre layers may remain ‘green’ despite early glaucomatous change. Because segmentation and color-coding algorithms are proprietary and opaque, clinicians cannot easily assess the performance of these systems across diverse demographics or biometric profiles.

These challenges underscore that RNFL colour maps should be interpreted as statistical summaries, not categorical diagnoses. In practice, glaucoma specialists already integrate OCT data with optic disc appearance and functional testing; nevertheless, the colour coding can carry psychological weight and influence diagnostic confidence, particularly among trainees and non-specialists. Greater transparency about the data underlying these classifications may help contextualise results and prevent over-reliance on automated outputs.

Meaningful progress can begin even before new datasets are built. Device manufacturers could display key metadata directly on OCT reports, such as the sample size, mean age, racial composition and axial length distribution of the normative database, allowing clinicians to judge how closely it aligns with their patient’s characteristics. Educational initiatives should reinforce that the colour map is one piece of a larger diagnostic puzzle, to be correlated with the B-scan for segmentation accuracy, with the visual field for functional corroboration and with the optic disc for structural confirmation.

Clinicians should also remain especially cautious when interpreting OCT in populations known to diverge from the reference sample, such as highly myopic individuals or those from ancestries under-represented in the original database. In such cases, longitudinal assessment, inter-eye symmetry and multi-modal correlation provide a more reliable picture than a single cross-sectional scan.

Many core OCT RNFL algorithms were developed more than a decade ago, before large-scale cloud aggregation and modern machine learning were feasible in clinical imaging. Although these early normative datasets were important milestones, OCT thresholds remain largely anchored to limited reference populations. This is a missed opportunity. With large, diverse datasets and validated analytic frameworks, OCT interpretation could shift from static categorisation toward earlier, individualised risk detection, enabling timelier intervention and reducing preventable glaucoma-related vision loss.

Long-term solutions will require collaboration across academia, industry and regulators. A global, multi-ethnic normative consortium could assemble a large-scale dataset encompassing thousands of healthy eyes, stratified by age, sex, ancestry, refractive status and disc morphology. Publicly accessible reference distributions would enable continual recalibration of device thresholds and allow harmonisation across vendors.

Manufacturers and oversight agencies could also implement algorithmic fairness audits, publishing false-positive and false-negative rates stratified by demographic variables, analogous to fairness reporting now encouraged in medical AI. Over time, the field should move toward device-agnostic normative standards, a shared statistical backbone akin to the DICOM standard, so that OCT outputs can be compared transparently across platforms and updated as new data emerge. Funding agencies and journals can reinforce these efforts by mandating disclosure of normative dataset composition in device approvals and published studies.

The RNFL colour map is one of ophthalmology’s most elegant visual tools, transforming complex quantitative data into an accessible clinical signal. Yet its clarity depends on the integrity of the reference populations that define ‘normal.’ When those populations are narrow, the resulting colours risk misleading more than they inform. By promoting transparency today and building inclusive normative frameworks for tomorrow, the field can ensure that the colours we trust truly reflect the diversity of the eyes we serve.
